# Academic-Service Partnerships in Nursing: An Integrative Review

**DOI:** 10.1155/2012/501564

**Published:** 2012-03-22

**Authors:** Judy A. Beal

**Affiliations:** School of Nursing and Health Sciences, Simmons College, Boston, MA 02115, USA

## Abstract

This integrative review summarizes currently available evidence on academic-service partnerships in the profession of nursing. More than 300 articles, published primarily in refereed journals, were accessed. Articles (110) were included in this review as they presented detailed and substantive information about any aspect of a nursing academic-service partnership. The majority were anecdotal in nature. Topics clustered around the following categories: *pre-requisites for successful partnerships, benefits of partnerships, types of partnerships*, and *workforce development* with its themes of *academic-practice progression* and *educational re-design.* Many examples of partnerships between academic and service settings were thoroughly described and best practices suggested, most often, however, without formal evaluation of outcomes. Nursing leaders in both settings have a long tradition of partnering with very little replicable evidence to support their efforts. It is critical that future initiatives evaluate the effectiveness of these partnerships, not only to ensure quality of patient outcomes but also to maximize efforts at building capacity for tomorrow's workforce.

## 1. Introduction

In November 2010, the Institute of Medicine (IOM) released its highly anticipated *Report on the Future of Nursing* [1]. This report called for major initiatives to redesign both nursing education and practice to better prepare nurses to lead during health care reform and beyond. In particular, the report outlined the need for increasing baccalaureate prepared nurses to 80% of the workforce, doubling the number of doctorally prepared faculty, removing scope of practice barriers, and disseminating successful collaborative improvement initiatives. Additionally, it recommended implementing nurse residency programs, ensuring that all nurses engage in lifelong learning, preparing and enabling nurses to lead change to advance health, and building an infrastructure for the collection and analysis of interprofessional health care workforce data [1]. It is clear that if we are to achieve these goals, academic and practice leaders must work together. Nurse leaders from both academic and service settings have a long history of collaborating [2–4], but now must do so more effectively and with greater sense of urgency than ever before [5]. 

A partnership is defined as an arrangement where parties agree to cooperate to advance their mutual interests [6]. In the profession of nursing, academic-service partnerships are most often defined as strategic relationships between educational and clinical practice settings that are established to advance their mutual interests related to practice, education, and research [7]. Although these alliances may be formally arranged, they are more typically informal in nature. More formal and structured relationships have been found to demonstrate greater levels of innovation and effectiveness [8]. 

Ironically, the twentieth century transition from hospital based training to academically-based nursing education contributed inadvertently but substantially to the development of a wide chasm between academic and service sectors [9]. The melding of nursing education and service dates back to the 17th century when the education of nurses was most often conducted in concert with religious traditions and communities [4]. Partnerships with physicians, medical colleges, and hospitals were the cornerstone of 18th and 19th century nursing education. While contemporary leaders have repeatedly advocated for nursing educators and clinicians to forge new collaborative bonds [2, 3, 5, 7, 10–13], the salience of their call has taken on new urgency in the wake of national health care reform's priorities of affordable quality care and provider accountability. The purpose of this integrative review of the existing literature on academic-service partnerships in nursing is to provide a foundation for future knowledge development by describing what is currently known about these ventures, and offering an agenda of priorities for moving forward.

## 2. Methods and Materials

An extensive review of the literature was conducted to synthesize current knowledge about academic-service partnerships in nursing. No other published report on this topic was found. The review included both empirical and conceptual articles published from 1990 through 2010. Data collection consisted of a search of the Cochrane Library, CINAHL, and MEDLINE databases using the following keywords: *nursing, nursing education partnerships, academic-service partnerships, academic-practice partnerships, *and *nursing education re-design*. Consistent with the methodological approach [14], references were also located through journal hand searching and the use of reference lists in retrieved articles. More than 300 articles, published primarily in refereed journals, were accessed. Eligibility criteria for inclusion in the review were English language; publication within the last twenty years; detailed and substantive information about any aspect of a nursing academic-service partnership.

## 3. Results

More than 300 citations were accessed. Of these, 110 met the criteria for inclusion, only nine of which reported on original research. Many examples of partnerships between academic and service settings were thoroughly described and best practices suggested, but most lacked formal evaluation of outcomes. An analysis of the main issues discussed in these articles clustered around the following categories: *pre-requisites for successful partnerships, benefits of partnerships, types of partnership, *and *workforce development *with its themes of* academic-practice progression *and *educational re-design. *Key points in each of these categories are described below. 

### 3.1. Pre-Requisites for Successful Partnerships

Many authors not only called for a recommitment to partnering but also identified key pre-requisites to developing and sustaining effective academic-service partnerships [15–34]. They acknowledged that the process of establishing such collaborative relationships can be both difficult and time consuming despite its rewards. Successful partnerships always start with self- and mutual-assessments of strengths and opportunities. From the very beginning, it is essential that each partner bring something valuable to the partnership [15–19]. Key elements of an effective partnership always included mutual trust; shared vision, commitment, and goals; mutual respect; recognition of opportunities and strengths; open and ongoing communication [20–27]. Specific strategies for developing and sustaining partnerships were written, formalized, and measureable goals and ongoing evaluation; strongly articulated institutional leadership support; the ability to take risks and tolerate ambiguity; structured accountability; institutionally shared resources; dedicated time; celebration of successes. While all of the articles reviewed either implied or explicitly stated the difficulties inherent in the process, all reported that if these key elements were in place, success and satisfaction were more likely, leading to sustainability of the partnership.

### 3.2. Benefits of Partnerships

According to Bleich et al. [3], who are considered thought leaders on the topic of academic-service partnerships in nursing, the benefits that were cited by the American Association of Colleges of Nursing (AACN) in 1990 [7] remain true today. These include strength and power in mutual goal setting, increased visibility and esteem for nursing's contribution to health care delivery, maximization of resources, enhanced opportunity for educators to remain current in practice, cost effective quality care and education of students and staff, increased research productivity, and development of patterns of excellence [7]. Other benefits of academic-service partnerships cited in the literature included improving organizational efficiencies, providing greater opportunities for innovations and new ways of thinking and doing [35, 36], and enhanced recruitment and retention [37].

### 3.3. Types of Partnerships

Academic-service partnerships have been widely discussed in the literature. These include partnerships with hospitals, community health, and public health agencies, nursing homes, schools, and governmental agencies. From early on in nursing's history, the focus of these partnerships was directed toward providing care to specific populations, educating students and staff, producing research, and addressing workforce issues in nursing. Faculty practice models and centers for research are two examples of partnerships that developed in the mid-20th century. Today, in the early 21st century, the majority of academic-service partnerships are focused on building workforce capacity. 

#### 3.3.1. Models of Faculty Practice

Faculty practice models, first seen in the United States in the 1950s [3, 38], emerged as innovative approaches to care for vulnerable populations, support training of advanced practice nurses, build a more educated workforce infrastructure, and advance nursing research [3, 12, 13, 15]. 

 One model of faculty practice was the academic nursing center, housed in the university and staffed by faculty. These centers provided cost-effective care to their neighboring communities [2, 39, 40]. While key challenges were mostly financial, or related to legal and regulatory issues [41], patient outcomes were consistently excellent [42]. 

While faculty practice had its early roots in academic nursing centers, other popular models allowed for joint appointments for both faculty in a practice setting and the practitioners in the university. In a critical review of 35 articles, Sawyer and colleagues [43] concluded that, by 2000, faculty practice had become an integral component of faculty role expectations at most schools of nursing. Successful faculty practice models are well described in the literature [44–67]. Authors consistently attributed this success to building a strong infrastructure to support the practice initiative, including administrative support for business, legal, and regulatory oversight [51, 55, 57, 60, 66]. Equally important were trust among partners [57], and a shared and well-articulated mission and vision [54, 55, 58, 60, 61]. 

The many benefits to faculty practice for all partners, regardless of model employed, included cost effectiveness, good patient outcomes, positive student learning, increased research productivity, and faculty satisfaction [49–64]. Additional benefits to the practice site were opportunities for staff to work with highly knowledgeable and engaged faculty members, and ability to recruit new graduates [63]. The communities served also benefited from the many practice innovations that were generated from the synergies developed between faculty, students, and providers [60]. Today, faculty practice models continue to provide opportunities for nurse practitioner faculty to maintain certification which is critical to accreditation of nurse practitioner academic programs [68].

#### 3.3.2. Centers for Research and Evidence Based Practice

While academic nursing centers and collaborative faculty practice collaborations were typically developed to provide patient care and student learning experiences, research collaborations between faculty and clinicians were often an unanticipated benefit. Development of partnerships for the primary purpose of advancing research and building an evidence-based practice began to emerge soon thereafter [3, 69–75]. In these partnerships, faculty has access to subjects for their research and clinicians have access to research experts and consultation. The outcomes of such partnerships included enhanced evidence-based practice, increased grant funding, and overall improvement in the “generation, dissemination, and application of knowledge for the improvement of nursing practice and patient outcomes” [70, page 114]. These research-focused partnerships have not only enhanced patient outcomes but also provided research training for a new generation of scholars.

### 3.4. Workforce Development Initiatives

The majority of academic-service partnerships in the past decade have focused on building professional nurse workforce capacity [76]. All of the recent workforce predictions point to a serious nursing shortage. The Robert Wood Johnson Foundation's 2010 report, for example, forecasts a deficit of “more than 260,000 registered nurses by 2025 unless we expand nursing education capacity quickly and dramatically” [76]. Published papers addressing this topic focused on anticipated problems associated with a nursing workforce inadequately prepared to lead and implement change, and the concomitant need for nursing education re-design. Authors proposed strategies to realign academic and service resources, with the goals of improving the quality of prelicensure education, building faculty capacity, and ensuring quality and safety of patient care. Two central themes that emerged in the literature review within this category include *academic-practice progression* and *educational re-design.*

#### 3.4.1. Academic-Practice Progression

In 2008, the Nursing Executive Center [77, 78] published its two-volume report entitled, “Bridging the Preparation-Practice Gap.” The first volume [77] of the report detailed the results of a survey of 400 nursing school directors and over 3500 hospital-based leaders on 36 competencies required of new graduates. The result indicated a need for better preparation of students on all 36 competencies. The top ten priorities for improvement included utilization of information technologies, rapport with patients and families, respect for diverse cultural perspectives, patient assessment, customer service, documentation, medication administration, patient advocacy, interdisciplinary team communication, accountability for one's actions, ability to work as a team member, and recognition of when to ask for help [77]. Importantly, the report noted polarized opposing views on practice readiness with “nearly 90 percent of academic leaders believing that their new graduate nurses are fully prepared to provide safe and effective care, compared to only 10 percent of hospital and health system executives” [78, page X]. The Board called for a more collaborative approach to close this significant gap, using strategies such as targeted clinical rotations, expert clinical instruction, and exceptional salient student experiences. 

Several approaches have been described to address this gap in preparation to practice readiness. The most wellknown is the senior capstone experience where students work one-on-one with a clinical nurse to care for assigned patients [79, 80]. Reports of other academic-service partnerships have focused on enhancing individual student experiences with vulnerable populations [75, 81–90]; with schools [91, 92], the elderly [93, 94], and in primary care settings [95, 96].

#### 3.4.2. Nursing Education Re-Design

At the same time that the Nursing Executive Center published its reports, data were emerging on how faculty shortages across the country were limiting student capacity [97, 98]. A more formalized educational re-design initiative was one approach to increase faculty capacity and to more effectively match the realities of clinical practice for today and tomorrow. A 2008 white paper [98] jointly commissioned by the RWJ Foundation, the Center to Champion Nursing in America (CCNA), and the U. S. Department of Labor, Employment, and Training Administration described innovative efforts underway to address the nursing faculty shortage. Four approaches to increasing nursing education capacity were described: (1) creating strategic partnerships to align and leverage stakeholder resources; (2) increasing nursing faculty capacity and diversity; (3) re-designing nursing education; (4) flexing policy and regulation [98]. Examples of innovations within these approaches included accelerated entry level BSN programs, centralized clinical placement initiatives, accelerated doctoral programs, adjunct clinical faculty training, the Robert Wood Johnson New Careers in Nursing Program, dedicated education units, nurse residency programs, Nursing Teacher Loan Forgiveness programs, and BSN in 10 legislation [98]. 

The development of accelerated postbaccalaureate nursing education programs was an early attempt at increasing the nursing workforce. According to the AACN [99], today there are more than 281 accelerated baccalaureate and 63 accelerated master's programs. While impressive in scope, these efforts alone cannot meet the future demands for a well-prepared and adequately numbered workforce. Furthermore, these programs were not designed as academic-service partnerships, and it has become increasingly apparent that nursing must work together to address the issues of workforce capacity. Reinhard and Hassmiller [100], along with Joynt and Kimball [98], described the collaboration between the RWJ Foundation and the American Association of Retired Persons (AARP), to create the Center to Champion Nursing in America (CNAA). The main emphasis of this center is raise awareness of the nursing shortage and to highlight innovative partnerships that are working to resolve this crisis. Between 2009 and 2010, the CNAA at AARP, the RWJ Foundation, the U.S. Department of Labor's Employment and Training Administration, and the U.S. Department of Health and Human Services' Health Resources and Services Administration (HRSA) cosponsored two national summits on nursing education capacity. Exemplars presented at these summits included public and private partnerships designed to prepare the future nursing workforce [76, 101]. Many of these are state-wide initiatives modeled after the Oregon Consortium for Nursing Education (OCNE). This consortium has completely redesigned the nursing curriculum and standardized nursing education in eight of the 15 community college nursing programs and the multicampus Oregon Health and Science University (OHSU) School of Nursing [17, 101, 102]. Other states with robust collaborations between public and private schools of nursing and their practice partners include California, Florida, New Jersey, Hawaii, Massachusetts, Michigan, Mississippi, New York, North Carolina Texas, and Virginia [76, 101]. Reported innovations include: analyses of the preparation-practice gap, seamless admissions and curricula, use of standardized patients, technology and simulation, the development of competencies for the nurse of the future, regional collaborations, retention strategies, centralized clinical placement systems, and faculty development initiatives. 

Some of this educational re-design work was developed as a result of AACN's 2003 support to establish the University Health System Consortium [103]. The consortium's purpose was to address the need to ensure a more educated workforce and a more effective transition into the professional role for baccalaureate graduates. Nurse executives and deans from hospitals and schools of nursing in academic health science centers in California, Iowa, Kentucky, Oregon, Tennessee, and Texas formed a joint task force that identified goals around the recruitment, retention, and expansion of baccalaureate students, successful transition through structured residency programs, and a professional and healthy work environment with differential salaries based on academic preparation. 

MacIntyre and colleagues [104] recommended five strategies to realign scarce resources of faculty and clinical placements with the goals of improving the quality of pre-licensure education, building faculty capacity, and ensuring quality and safety of patient care. They argued (1) that faculty and practice leaders must reconceptualize nursing student and staff relationships as well as the role of faculty by encouraging experienced staff nurses to become more involved in the education of students. (2) Experienced faculty members from the university could then become mentors to novice staff educators who in turn may become excited about pursuing the faculty role. Furthermore, they recommended (3) that schools of nursing develop resources to prepare the novice educators for the teaching role. They encouraged faculty (4) to think about alternative clinical sites and experiences with their practice partners and (5) to build the evidence-based content of the curriculum. As changes such as these require full partnership, they must be based on a sound methodological approach and transparency [105]. If effective, MacIntyre and colleagues [104] concluded that such an approach would increase workforce capacity. 

Dedicated education units (DEU) were designed as partnerships and resonate with the suggestions made by MacIntyre and colleagues. One of the first articles to describe the DEU came from Flinders University in Australia and was published in 1999 [106]. A dedicated education unit is a “partnership of nurse executives, staff nurses, and faculty who transform patient units into environments of support for nursing students and staff nurses while continuing the critical work of providing care…” [107, page 31]. The DEU model employs well-prepared RN clinicians as educators for nursing students. Academic faculty serve as role models and mentors to these scholar clinicians. While a number of models have been described [106–112], only a few studies have been published to date evaluating outcomes of this recent innovation [106, 108, 111, 112]. Of these, all concluded that the dedicated education increased educational capacity as well as student and staff satisfaction, and was a good source for clinical placements.

The development of the clinical nurse leader (CNL) role and supporting curriculum is another recent example of academic-service partnership that addresses the preparation-practice gap. The AACN proposed the CNL role in 2003 after consulting with an extensive group of leaders from a variety of settings across the healthcare delivery system. The CNL is a provider and a manager of care at the point of care to individuals and cohorts [113]. The master's level curriculum is based on values and core competencies identified by the Nursing Executive Center [97, 98]. The curriculum also addresses concerns voiced by the Institute of Medicine [114, 115] regarding the fragmentation of healthcare delivery that contributes significantly to the high error rate and decreased quality in patient care. Starting in 2004, 77 academic-practice partners began piloting several CNL demonstration projects. Today, there are more than 108 academic partners and 210 practice partners, a CNL certification examination, and hundreds of CNL graduates across the country. The Department of Veterans Affairs has committed to implementing the CNL role in all VA medical centers by 2016 [116]. 

As is typical with the development of CNL programs, nursing leaders from three New Jersey healthcare organizations partnered with the College of New Jersey to develop their curriculum. They worked together to implement the program with representatives from both practice and the academic settings involved in teaching the students [117]. A focus on clinical relevance was a key element in the curriculum which addressed prior concerns about the preparation-practice gap [97, 98].

Smith and Dabbs [118] published one of the few studies to include patient outcomes in their formative and summative evaluation methodology to assess outcomes of a precursor to the CNL model on a 46-bed medical-surgical unit. A year after implementation, falls with injury were decreased by 67% and pressure ulcer rates decreased to 0%. Patient satisfaction increased and staff turnover decreased. The average length of stay decreased from 4.22 to 3.45 days on the pilot unit and patient satisfaction with discharge planning improved.

## 4. Discussion and Conclusions

While the literature is replete with descriptions of numerous academic-service partnerships that have developed over the past twenty years, objective evidence for the success of these relationships is limited at best. While much informal evaluation has occurred, very few of the innovations described have been formally studied. Generalizability of research published to date is limited by small, nonrepresentative samples in single locations followed over relatively short time periods. 

As academic and service partners move forward to design and implement changes to prepare the next generation of professional nurses for the realities of a changing healthcare delivery landscape, both process and outcome evaluations must be incorporated in the planning. Both research and quality improvement methodologies may be useful, depending on the proposed innovation. The key point is that measurement is essential to determine the short- and long-term effectiveness and the efficiency of these initiatives. We will need to know, for example, the kind and number of staff and faculty resources required to ensure success, and their associated costs. The effect on patient outcomes must also be considered, as well as staff and faculty outcomes, such as readiness for practice and effects on workloads, job satisfaction, recruitment, and retention.  Table 1 outlines a more comprehensive list of suggested outcomes to measure partnership success. 

Despite the limitations of the extant literature, this integrative review has highlighted a number of successful or promising academic-service partnerships and associated benefits perceived by the innovators. Thought leaders have also described the prerequisites that should frame future partnerships. The IOM's call [1] is urgent and compelling. Nursing leaders are stepping up to the plate on the local, regional, and national levels to respond to the inherent challenges. Schools of Nursing and their practice partners continue to develop new approaches to solve the problems of the lack of clinical faculty and clinical placements, as well as the nursing shortage. States and regions are responding to the call from the RWJ Foundation to establish Regional Action Coalitions. Leaders from the AACN, the American Organization of Nurse Executives, and the Association of State and Territorial Directors of Public Health Nursing have partnered to form a national task force to identify best practices in academic-service partnerships. The nursing profession eagerly awaits the results from these impressive partnership activities. The time is right to move forward by building together on past successes for both the good of our patients and the continuing development of our professional capacity.

## Figures and Tables

**Table 1:** Suggested outcome measures of effective partnerships.

Outcome measures for effective academic-service partnerships at the individual partner level	Expected outcomes
	The number of quality clinical placements will increase and diversify
	The number of qualified clinical faculty recruited from clinical partnership sites will increase
	The opportunities for shared experiences (research, practice projects, shared teaching, DEUs, etc.) between faculty and clinical staff will increase
	The number of students enrolled will increase along with the quality of students accepted
	Academic progression policies will support excellence
	Student retention will be increased
	Student performance on NCLEX-RN will increase
	Student employment rates post graduation will increase
	Orientation time for new graduates will decrease
	Recruitment and orientation costs to service organizations will decrease
	Retention rates for new graduates will increase
	Patient safety and quality indicators of success will increase
	The percentage of nurses who return for advanced degrees will increase
	Research Productivity will increase
	The percentage of nurses who become leaders within their institutions and beyond will increase
	The percentage of nurses who become politically active will increase
	Satisfaction of students, staff, faculty, and employers will increase

Outcome measures for effective academic-service partnerships at the regional level	The numbers of regional action coalitions will increase and results of their work will be disseminated nationally

Outcome measures for effective academic-service partnerships at the individual level	The AACN-AONE task force on academic-practice partnerships will take a national role in promoting a national level discussion of and implementation plan on best practices and implementation strategies
